# Pre-Clinical Investigation of Keratose as an Excipient of Drug Coated Balloons

**DOI:** 10.3390/molecules25071596

**Published:** 2020-03-31

**Authors:** Emily Goel, Megan Erwin, Claire V. Cawthon, Carson Schaff, Nathaniel Fedor, Trevor Rayl, Onree Wilson, Uwe Christians, Thomas C. Register, Randolph L. Geary, Justin Saul, Saami K. Yazdani

**Affiliations:** 1 Department of Mechanical Engineering, University of South Alabama, Mobile, AL 36688, USA; emily.turner.ann@gmail.com (E.G.); megan.m.erwin@vanderbilt.edu (M.E.); clairecawthon@gmail.com (C.V.C.); carson.schaff@gmail.com (C.S.); ntfedor@gmail.com (N.F.); trevorerayl@gmail.com (T.R.); onreewilson@gmail.com (O.W.); 2 iC42 Clinical Research and Development, Department of Anesthesiology, University of Colorado; Aurora, CO 80045, USA; Uwe.Christians@ucdenver.edu; 3 Department of Vascular Surgery, Wake Forest School of Medicine, Winston-Salem, NC 27157, USA; register@wakehealth.edu; 4 Department of Pathology, Wake Forest School of Medicine, Winston-Salem, NC 27157, USA; rgeary@wakehealth.edu; 5 Department of Chemical, Paper and Biomedical Engineering, Miami University, Oxford, OH 45056, USA; sauljm@miamioh.edu; 6 Department of Engineering, Wake Forest University, Winston-Salem, NC 27101, USA

**Keywords:** Keratose, drug-coated balloon, paclitaxel, drug delivery, pre-clinical, peripheral arterial disease, endovascular

## Abstract

Background: Drug-coated balloons (DCBs), which deliver anti-proliferative drugs with the aid of excipients, have emerged as a new endovascular therapy for the treatment of peripheral arterial disease. In this study, we evaluated the use of keratose (KOS) as a novel DCB-coating excipient to deliver and retain paclitaxel. Methods: A custom coating method was developed to deposit KOS and paclitaxel on uncoated angioplasty balloons. The retention of the KOS-paclitaxel coating, in comparison to a commercially available DCB, was evaluated using a novel vascular-motion simulating *ex vivo* flow model at 1 h and 3 days. Additionally, the locoregional biological response of the KOS-paclitaxel coating was evaluated in a rabbit ilio-femoral injury model at 14 days. Results: The KOS coating exhibited greater retention of the paclitaxel at 3 days under pulsatile conditions with vascular motion as compared to the commercially available DCB (14.89 ± 4.12 ng/mg vs. 0.60 ± 0.26 ng/mg, *p* = 0.018). Histological analysis of the KOS–paclitaxel-treated arteries demonstrated a significant reduction in neointimal thickness as compared to the uncoated balloons, KOS-only balloon and paclitaxel-only balloon. Conclusions: The ability to enhance drug delivery and retention in targeted arterial segments can ultimately improve clinical peripheral endovascular outcomes.

## 1. Introduction

Drug-coated balloons (DCBs) represent a new therapeutic approach to treat peripheral arterial disease (PAD) [1,2,3,4,5]. In the United States, PAD affects more than eight million people, with an annual cost of roughly $21 billion [6]. Traditionally, endovascular treatment of PAD has been performed by balloon angioplasty or the placement of a permanent metallic stent [7,8]. However, results are poor, with 50–85% of patients developing hemodynamically significant restenosis (re-occlusion), and 16–65% developing occlusions within 2 years post-treatment [9,10]. The use of anti-proliferative drugs in combination with bare metal stents, i.e., drug-eluting stents (DES), was a major breakthrough and highly successful in treating coronary artery disease [11,12]. However, stents have shown very poor clinical outcomes in treating PAD, as they are subjected to biomechanical stress and severe artery deformation (twisting, bending, and shortening), leading to high fracture rates (up to 68%) and restenosis [13].

DCBs, which were FDA-approved for the treatment of PAD in late 2014, provide a new therapeutic approach for interventionalists to practice a ‘leave nothing behind’ procedure, preserving future treatment options DCBs are angioplasty balloons directly coated with an anti-proliferative therapeutic drug and an excipient (drug carrier) [1,14,15,16,17,18]. The excipient enhances the adhesion of the drug to the balloon surface, increases the stability of the drug coating during handling and delivery, and maximizes drug retention to the targeted arterial segment. [18,19,20,21,22,23,24] Current DCBs excipients include polysorbate and sorbitol, urea, polyethylene glycol (PEG) and butyryl-tri-hexyl citrate (BTHC). The rationale for the selection of these various excipients varies. For example, excipients such as polysorbate and PEG are known cosolvents of paclitaxel [25,26], which can alter the vessel interaction of the drug with the DCB device. Conversely, urea acts to increase paclitaxel release at the lesion [18] and PEG has been shown to bind to hydroxylapatite, a primary component of calcified atherosclerotic lesions [17,19,23,24], thereby improving local pharmacodynamics.

However, more recent pre-clinical studies have demonstrated the potential of DCB excipients to embolize and travel downstream to distal tissue post-treatment [27,28]. As peripheral arteries undergo severe mechanical deformation, excipients should aid in maintaining drug residency on the luminal surface, in particular at the early time phase, prior to the buildup of tissue, following delivery onto the luminal surface of the artery. Therefore, novel excipients that are capable of maintaining drug residency while minimizing downstream or off-target effects are needed. Keratins are a class of proteins that can be derived from numerous sources, including from human hair. Keratins have been shown to achieve the sustained release of small-molecule drugs and growth factors [29,30]. Further, keratin films have been reported for use in vascular grafts to reduce thrombosis, suggesting their utility in cardiovascular applications [31]. The goal of this study was thus to examine the ability to use an oxidized form of keratin (known as keratose (KOS)) as a new drug carrier excipient to aid in the delivery and retention of the anti-proliferative drug, paclitaxel. Specifically, the mobility, retention and biological impact of a KOS–paclitaxel-coated DCB was determined using *ex vivo* and *in vivo* models. 

## 2. Results

### 2.1. Ex Vivo Results

**Figure 1:**
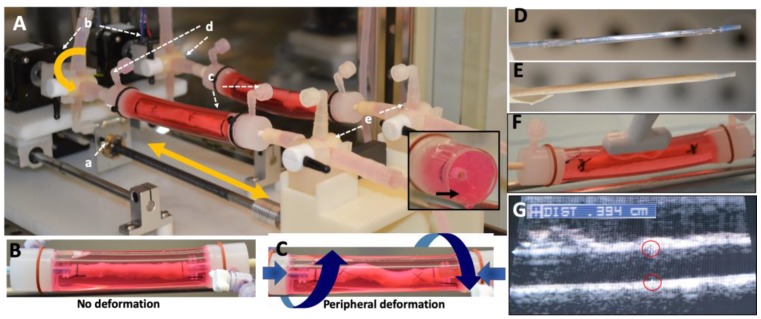
Schematic illustration of the novel peripheral simulating bioreactor system. (**A**) Two servos (a and b) provide axial deformation and twisting, respectively. Servo (a) moves the harvested arteries (c) forward and backward in a linear motion. Servo b rotates the artery by degrees. The three-way values (d and e) are used to introduce flow and pressure to the artery. The artery is surrounded by matrigel, mimicking external tissue, providing support during vascular movement (insert, black arrow). (**B**,**C**) Harvested arteries under pulsatile (no deformation) and pulsatile conditions with peripheral deformation. (**D**,**E**) Gross photos of an uncoated balloon and a keratose–paclitaxel coated balloon. (**F**,**G**) Diameter measurements performed by ultrasound on the harvested artery.

**Figure 2:**
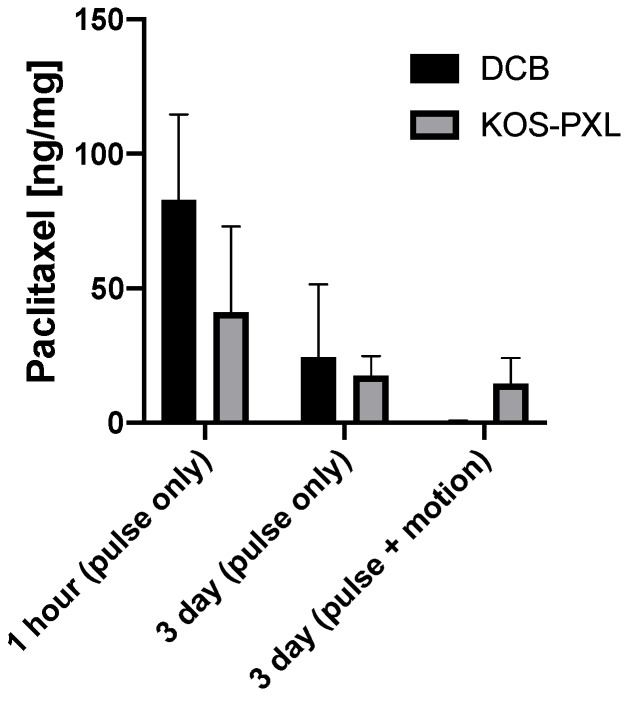
Paclitaxel levels of the drug-coated balloon (DCB) and the keratose–paclitaxel (KOS-PXL)-coated balloon arterial segments undergoing pulsatile flow conditions versus pulsatile flow conditions with vascular motion.

The non-coated angioplasty balloons were successfully coated with the KOS–paclitaxel mixture (Figure 1D,E). To determine the impact of vascular deformation on DCB retention, both the KOS–paclitaxel DCB and the commercially available DCB were tested under only physiological pulsatile conditions (no twisting or shortening) and physiological pulsatile conditions with vascular deformation conditions. The pulsatile flow conditions consisted of pressures ranging from 70 to 120 mmHg with a mean flow rate of 120 mL/min at 60 beats per minute. The vascular deformation conditions consisted of the artery shortening 10% in the axial direction and twisting of the artery at 15°/cm. The frequency of the artery twisting and shortening was 0.05 Hz (3 cycles/min). All DCBs were inserted through a 6 Fr sheath into the closed-circulatory system under the physiological pulsatile conditions. The treated sections of the artery were marked during inflation of the DCBs. It is noted that vascular deformation (twisting and shortening) occurred following 4 h of physiological pulsatile conditions of DCB deployment. At timepoints of 1 h and 3 days, the treated section of the arteries were removed and analyzed for arterial tissue paclitaxel concentration (Figure 2). There was a reduction in arterial paclitaxel levels from 1 h to 3 days post-treatment for both the KOS–paclitaxel and the commercially available DCB under physiological pulsatile conditions (3 days—pulse only: KOS-PXL: 17.56 ± 7.19 ng/mg vs. commercial DCB: 24.44 ± 27.03, *p* = 0.96, Table 1). However, under pulsatile and vascular deformation, paclitaxel was significantly retained within the treated artery (3 days—pulse and vascular deformation: KOS-PXL: 14.89 ± 4.12 ng/mg vs. commercial DCB: 0.60 ± 0.26, *p* = 0.018). 

### 2.2. Histomorphometric Results

**Figure 3:**
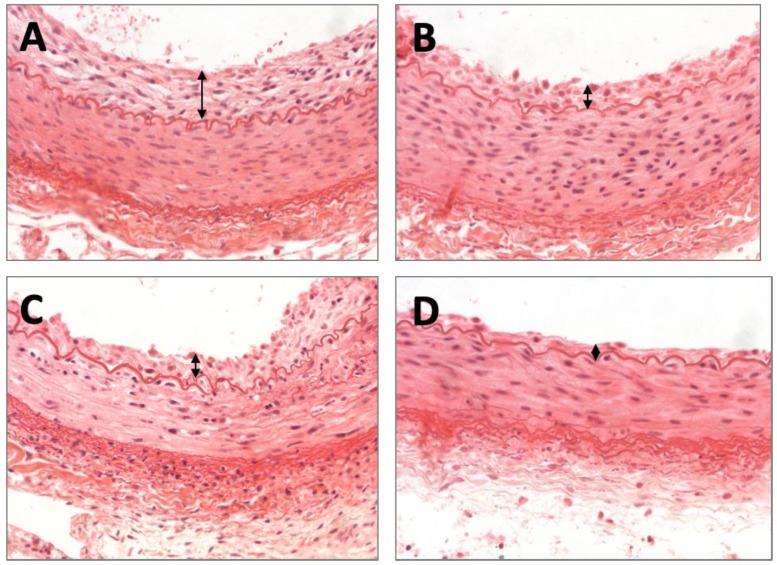
Representative images of the arterial response following the varying treatment groups. H&E staining demonstrated neointimal growth for (**A**) the uncoated balloon group, (**B**) keratose-only coated balloon, (**C**) paclitaxel-only coated balloon and (**D**) keratose–paclitaxel coated balloon at 7 days. Neointimal growth is marked by double-arrow heads.

Following *ex vivo* studies, *in vivo* studies were performed using the rabbit ilio–femoral injury model to determine the impact of the KOS excipients on vascular remodeling. The animals were treated with a KOS-paclitaxel (*n* = 4), KOS-only balloon (*n* = 4), paclitaxel-only balloon (*n* = 4) or an uncoated balloon (*n* = 4). All arteries were treated successfully without any signs of dissection or thrombosis, and all animals survived the duration of the study. At 7 days, morphometric analysis demonstrated similar area measurements, including the EEL, IEL, lumen and media, for all treatment groups (Table 2). Neointimal thickness was significantly different between the varying groups (no coating: 0.10 ± 0.011 mm vs. KOS-only: 0.069 ± 0.022 mm vs. PXL-only: 0.066 ± 0.018 mm vs. KOS-PXL: 0.53 ± 0.003 mm, *p* = 0.005, Figure 3). Although percent area stenosis was the least in the KOS-PXL group, differences between the group were non-significant (no coating: 10.88% ± 4.52% vs. KOS-only: 9.99% ± 3.78% vs. PXL-only: 7.92% ± 3.84% vs. KOS-PXL: 6.80% ± 2.74%, *p* = 0.45). 

Histological analysis demonstrated minimal injury for all groups at 7 days with the greatest endothelial cell loss in the KOS–paclitaxel treated arteries (no coating: 0.25 ± 0.50 vs. KOS-only: 0.00 ± 0.00 vs. PXL-only: 0.25 ± 0.58 vs. KOS-PXL: 1.50 ± 0.58, *p* = 0.013). Inflammation was minimal for all groups and there was a trend towards greater SMC loss in the KOS–paclitaxel group (no coating: 0.00 ± 0.00 vs. KOS-only: 0.00 ± 0.00 vs. PXL-only: 0.25 ± 0.50 vs. KOS-PXL: 1.00 ± 0.82, *p* = 0.45). No aneurysmal dilatation or thrombosis was observed in any treated artery.

## 3. Discussion

This study was designed to evaluate the use of keratose as a novel excipient for peripheral applications and, specifically, to determine the feasibility of the keratose excipient to retain paclitaxel under peripheral vascular mechanical environments. This was accomplished by developing a novel vascular-simulating *ex vivo* flow system and testing in a clinically relevant pre-clinical model. Furthermore, the vascular biological response to the keratose excipient was also investigated in the pre-clinical model. The *ex vivo* model arterial drug concentration results demonstrated that keratose significantly improves the retention of paclitaxel as compared to a commercially available DCB. Histomorphometric results of rabbit arteries treated by keratose demonstrated the safety and efficacy of the excipient in the delivery of paclitaxel. Overall, these results demonstrate the potential of the keratose as a DCB excipient for peripheral applications.

Drug-coated balloons are the next-generation treatment for PAD. Approved in the US since late 2014, DCB represented a shift in the approach to treating peripheral artery disease. While the DES provides a scaffold for long-term drug release, DCBs are limited in the time they can interact with the target lesion (~30 s to 2 min). Therefore, a major goal of any excipient is to support the retention of the therapeutic agent to the arterial wall surface, even under vascular deformation conditions. In two recent studies, the embolization of release particulates from all currently FDA-approved DCB coatings was investigated [27,28]. Twenty-eight days post-delivery, their results also demonstrated evidence of distal embolization, including embolic crystalline material, in downstream tissue. Remarkably, pharmacokinetic analysis of the distal tissue showed similar or higher levels of paclitaxel concentration as compared to the arterial treatment site, in particular for the IN.PACT DCB. These results indicate the mobility of the DCB coating following deployment, although, to date, no studies have directly investigated the impact of vascular deformation on DCB performance.

The vascular-mimicking *ex vivo* system, to our knowledge, is the first system that can evaluate the acute drug-loading of arteries treated by endovascular devices under pulsatile and vascular deformation conditions using explanted pig arteries. Our testing of the KOS coating was performed under vascular deformation conditions of 10% artery shortening, 15°/cm twisting at a frequency of 0.05 Hz (3 cycles/min). These conditions were selected to replicate the human periphery motion of the femoral artery (shortening lengths of 7% and twisting at 11.5°/cm) and the popliteal–tibial artery motion (shortening of 15% and twisting at 19.9°/cm) [32,33,34,35]. The frequency of the peripheral movement will be 0.06 Hz (5184 cycles/day or 3.6 cycles/min), which is based upon the average steps per day of adults in the US [36]. Our results indicated that the KOS coating maintained paclitaxel tissue levels under physiological pulsatile and vascular motion conditions 3 days post-delivery.

**Figure 4:**
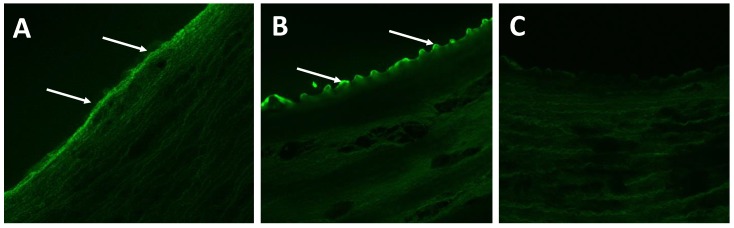
Representative confocal images arterial segments following keratose delivery. Confocal microscopy confirmed the presence of the keratose at (**A**) 1 h and (**B**) 3 days under peripheral deformation conditions. (**C**) Negative control depicts the lack of tissue autofluorescence during confocal imaging.

To further evaluate the DCB coating, we fluorescently tagged (NHS-Fluorescein, Thermo Scientific) the KOS to visualize the presence of the coating acutely (1 h) and three days post-delivery in arteries undergoing vascular deformation. The presence of the KOS was confirmed by confocal microscopy (Figure 4). The mechanism by which this process occurs is not fully elucidated in these studies. In drug-release experiments with small molecule drugs such as ciprofloxacin from hydrogel (rather than coating) forms of KOS, we have demonstrated that the rate of drug release correlates with the degradation rate of the hydrogel material [30]. We note that this degradation process does not refer to the breaking of peptide (amide) bonds in the keratin, but rather the dissolution of the keratin hydrogels. This correlation between drug release and KOS dissolution (or degradation) suggested an interaction between keratin and the drug. In the case of ciprofloxacin, this was found to be associated with electrostatic interactions. While the physiochemical characteristics of paclitaxel are different than ciprofloxacin, such interactions (or others, such as hydrophobic interactions) could be in play and are an area for further study. 

This previous finding of an interaction between KOS and small molecule drugs is noteworthy due to the findings of paclitaxel retention in the vessel at 3 days with vascular motion compared to DCB (Figure 2) and the presence of (fluorescently labeled) KOS on the vascular walls (Figure 4). That is, it is possible that paclitaxel remains associated with the KOS in a manner not possible with other synthetic polymers (e.g., PEG) or other (e.g., urea) excipients due to the properties of keratin. In particular, KOS has been shown to contain RGD and other integrin-binding sequences which may allow it to bind to the vascular cells [37,38]. Thus, KOS may have a unique ability to associate with the lumen through integrin-binding with vascular cells while simultaneously retaining the paclitaxel through electrostatic or hydrophobic interactions.

While it is well-recognized that arterial repair after balloon injury occurs more rapidly in animals than in humans, animal models still hold a predictive value for the observation of biological effects that may be associated with drug delivery [39]. In this study, histopathologic evaluation of the KOS–paclitaxel DCB, along with uncoated balloons, KOS-only balloons and paclitaxel-only DCBs were performed in a rabbit ilio–femoral injury model, which has been shown to be an appropriate model for the evaluation of endovascular devices [40,41,42,43]. Overall, the morphometric results demonstrated minimal neointimal growth, as percent area stenoses were less than eleven percent for all groups at the 7-day time point. These results were expected as, in general, peripheral rabbit arteries appear to be resistant to the development of aggressive neointimal growth with mild balloon to artery ratio (1.1–1.2:1), especially with plain balloon angioplasty [39,43]. Furthermore, as expected, injury scores were mild, ranging from 0.50 to 1.13 in all groups. However, by histologic evaluation, the safety and effectiveness of the KOS–paclitaxel DCB was still evident, based on vascular remodeling and healing. Specifically, neointimal thickness was significantly reduced in the KOS–paclitaxel DCB treatment group (no coating: 0.10 ± 0.011 mm vs. KOS-only: 0.069 ± 0.022 mm vs. PXL-only: 0.066 ± 0.018 mm vs. KOS-PXL: 0.53 ± 0.003 mm, *p* = 0.005). Importantly, the endothelization score was significantly reduced in the KOS–paclitaxel treated arteries, indicative of drug retention (Table 1). Additionally, there was a trend towards a lower neointimal area and higher loss of smooth muscle cells (SMCs) in the KOS–paclitaxel DCB group as compared to all others, indicative of drug effect (no coating: 0.00 ± 0.00 vs. KOS-only: 0.00 ± 0.00 vs. PXL-only: 0.25 ± 0.0.50 vs. KOS-PXL: 1.00 ± 0.82, *p* = 0.081). Overall, the *in vivo* data demonstrate the safety of the keratose coating and a reduction in neointimal growth by the keratose–paclitaxel DCB. 

While our results support the concept of a keratose coating to deliver anti-proliferative drugs to arterial segments, the study was limited to a healthy animal model and thus did not take into consideration diseased arteries, as observed in patients with PAD. For the *ex vivo* studies, further characteristic testing of paclitaxel delivery via the drug-coated balloon is warranted to quantify the amount of drug remaining on the balloon following delivery and to quantify circulating paclitaxel levels. We also recognize that human lesions are more complex and often include fibrosis, calcification, hemorrhage and, in most cases, require de-bulking using balloons and atherectomy devices, which may alter drug transfer and retention. While preclinical studies involving healthy arteries are the standard model to determine the arterial time drug concentration of cardiac and stent-based intervention devices, further improvement may be found with a KOS-paclitaxel coating in injury models.

## 4. Materials and Methods 

### 4.1. Keratose-Coated Balloons

The KOS-coated balloons were prepared as previously described [44,45]. Briefly, paclitaxel (LC Laboratories, Woburn, MA, USA) was prepared by dissolving paclitaxel in absolute ethanol followed by sonication at a final concentration of 40 mg/mL. Keratose (KeraNetics LLC, Winston-Salem, NC, USA) solution was prepared by dissolving lyophilized keratose in iohexol (GE Healthcare, Little Chalfont, UK) at a 6% weight-to-volume ratio in. An in-house air spray coating method was used to deposit keratose and paclitaxel in a layered approach on uncoated angioplasty balloons (Abbott Vascular, Abbott Park, IL, USA) [45]. Coated balloons were then sterilized by UV irradiation. 

### 4.2. Peripheral-Simulating Bioreactor

The peripheral-simulating bioreactor was designed to shorten and twist two harvest porcine carotid arteries subjected to pulsatile flow conditions (Figure 1A). The overall system (46 × 19 × 19 cm) was designed to fit inside of a standard CO_2_ incubator by arranging the arteries in a parallel configuration. The system utilizes one stepper motor per artery for rotational motion and one stepper motor for the translational motion of both arteries. Custom connectors were machined to mount the arteries to the stepper motors. The motion of the stepper motors was measured using rotary encoders (CUI AMT11, Tualatin, OR, USA) mounted on the shaft of each motor. An Arduino microcontroller with two motor shields was used to control the motors along with an LCD keypad module to provide an intuitive user experience, displaying time and cycles remaining for each test and providing physical inputs to start, stop, input artery length and the duration of testing. 

The carotid arteries, positioned within the vascular-simulating bioreactor, were harvested from large pigs (250–350 lbs.) from a local abattoir and transferred in sterile PBS with 1% antibiotic-antimitotic (Gibco, Grand Island, NY, USA). The arteries were then rinsed in sterile PBS in a culture hood and trimmed. Eight-cm-long segments were cut and tied with sutures onto fittings within the *ex vivo* setup. The circulating medium consisted of the system made up of Dulbecco’s modified eagle’s medium containing 10% fetal bovine serum and 1% antibiotic–antimycotic. 

### 4.3. Ex Vivo DCB Testing and Arterial Time Drug Concentration

Prior to any vascular motion (twisting and shortening), all arteries were subjected to pulsatile flow for 1 h, as defined by a custom LabVIEW program as previously described [42] The pressure was monitored via a pressure catheter transducer (Millar Instruments, Houston, TX). Flow was monitored by an ultrasonic flow meter. Following this pre-conditioning phase, the vessel diameter was measured by ultrasound (Figure 1F,G). Harvested arteries were then treated by either the KOS–paclitaxel-coated balloon or a commercially available DCB (In.PACT Admiral DCB, Medtronic, Santa Rosa, CA, USA). The delivery pressure of the DCB was determined by the manufacturers’ specification at a 10–20% overstretch. At timepoints of one hour and three-days, flow was ceased, and the treated portion of the vessel was removed. Excised vessels were flash frozen, stored at −80 °C and shipped on dry ice to iC42 Clinical Research and Development (Aurora, CO, USA) for the quantification of arterial paclitaxel. Quantification of arterial paclitaxel levels was performed using a validated high-performance liquid chromatography (HPLC)-electrospray ionization- tandem mass spectrometry assay (LC-MS/MS) [44,45,46]. In brief, the LC-MS/MS system was a series 1260 HPLC system (Agilent Technologies, Santa Clara, CA, USA) linked to a Sciex 5000 triple-stage quadrupole mass spectrometer (MS/MS, Sciex, Concord, ON, USA) via a turbo-flow electrospray ionization source. The artery tissue samples were homogenized using an electric wand homogenizer (VWR 200, VWR International, Radnor, PA, USA) after the addition of 1 mL of phosphate buffer. Eight hundred (800) μL of 0.2 M ZnSO_4_ 30% water/70% methanol *v*/*v* protein precipitation solution containing the internal standard (paclitaxel-D_5_, 10 ng/mL) was added. Samples were vortexed for 5 min, centrifuged (16,000, 4 °C, 15 min) and transferred into glass HPLC vials. Study samples were diluted as necessary for detector signals to fall within the dynamic MS/MS detector range. One hundred (100) μL of the samples was injected onto a 4.6 × 12.5 mm extraction column (Eclipse XDB C8, 5 μm particle size, Agilent Technologies, Palo Alto, CA, USA). Samples were washed with a mobile phase of 15% methanol and 85% 0.1% formic acid using a flow of 3 mL/min. The temperature for the extraction column was 65 °C. After 1 min, the switching valve was activated and the analytes were eluted in the backflush mode from the extraction column onto a 150 × 4.6 mm analytical column (Zorbax XDB C8, 3.5 µm particle size, Agilent). The analytes were eluted from the analytical column using a gradient of methanol/acetonitrile (1/1 *v*/*v*) plus 0.1% formic acid (solvent B) and 0.1% formic acid in HPLC grade water (solvent A). The MS/MS was run in the positive multi-reaction mode and the following ion transitions were monitored: *m*/*z* = 876.6 [M + Na]^+^ → 308.2 (paclitaxel) and *m*/*z* = 881.6 [M + Na]^+^ → 313.1 (the internal standard paclitaxel-D_5_). Paclitaxel tissue concentrations were calculated based on paclitaxel/paclitaxel-D_5_ peak area ratios using a quadratic regression equation with 1/x weighting. The range of reliable response was 0.5–100 ng/mL tissue homogenate. Inter-day imprecision was less than 15% and accuracy was within 85–115% of the nominal concentrations. There were no significant matrix interferences, carry-over or matrix effects. For more details, please see the aforementioned publications [42,44,45].

### 4.4. Rabbit Injury Model

This study was approved by the Institutional Animal Care and Use Committee and conformed to the position of the American Heart Association on use of animals in research. The experimental preparation of the animal model has been previously reported [42,46]. Under fluoroscopic guidance, eight anesthetized adult male New Zealand White rabbits underwent endothelial denudation of both iliac arteries using an angioplasty balloon catheter (3.0 × 8 mm). Subsequently, arteries were treated by either KOS–paclitaxel (3.0 × 15 mm), KOS-only balloon (3.0 × 15 mm), paclitaxel-only balloon (3.0 × 15 mm), or an uncoated balloon (3.0 × 15 mm) at a delivery pressure of 8 atm for two minutes. Anti-platelet therapy consisted of aspirin (40 mg/day) given orally 24 h before catheterization, with continued dosing throughout the in-life-phase of the study, while single-dose intra-arterial heparin (150 IU/kg) and lidocaine were administered at the time of catheterization. The animals survived for 7 days and subsequent histological evaluations were performed. 

### 4.5. Arterial Sections

Following the duration of the study, animals were anesthetized and euthanized, and the treated artery segments were removed based on landmarks identified by angiography. The arteries were perfused with saline and formalin-fixed under physiological pressure prior to removal. The segments were stored in 10% formalin at room temperature and then processed to paraffin blocks, sectioned, and stained with Hematoxylin and Eosin (H&E) or Verhoeff’s elastin stain (VEG).

### 4.6. Histomorphometric Analysis

Histological sections were digitized and measurements performed using ImageJ software (NIH). Cross-sectional area measurements included the external elastic lamina (EEL), internal elastic lamina (IEL), and lumen area of each section. Using these measurements, the medial area, neointimal area and percent area stenosis were calculated as previously described [46,47]. 

Morphological analysis was performed by light microscopy using a grading criterion as previously published [46,47]. The parameters assessed included intimal healing as judged by injury, endothelial cell loss and inflammation. The medial wall was also assessed for drug-induced biological effect, specifically looking at smooth muscle cell loss. These parameters were semi-quantified using a scoring a system of 0 (none), 1 (minimal), 2 (mild), 3 (moderate) and 4 (severe) as previously described [47].

### 4.7. Statistical Analysis

Results are reported as mean ± standard deviation. Data were compared with analysis of variance (ANOVA) using GrapPad Prism 7 (GraphPad Software, La Jolla, CA, USA). The comparison of quantitative data of multiple groups was performed by Tukey’s multiple comparisons post hoc test. Significance is reported as *p* < 0.05. 

## 5. Conclusions

This study provides evidence of the use of keratose as an excipient for peripheral applications. The *ex vivo* results showed a potential benefit of the coating to minimize the adverse impact of vascular motion on drug mobility and favorable biological response in the pre-clinical model. Additional studies are warranted to further demonstrate the safety and efficacy profile of the keratose coating in larger animal models and longer durations. Overall, this approach has the potential to improve interventional outcomes and quality of life of millions of patients suffering with PAD.

## Figures and Tables

**Table 1:** *Ex vivo* arterial drug concentration measurements of treated arteries.

	KOS-PXL	Commercial DCB	*p* Value
**Time Points**	ng/mg	ng/mg	
1 h (pulse only)	41.01 ± 32.11	82.88 ± 31.81	0.30
3 day (pulse only)	17.56 ± 7.19	24.44 ± 27.03	0.96
3 day (pulse + vascular motion)	14.89 ± 4.12	0.60 ± 0.26	0.018

**Table 2:** Summary of the morphometric and histological measurements in the rabbit iliac–femoral injury model.

	No Coating	KOS-only	PXL-only	KTO-PXL	*p* Value
**Morphometric Measurements**					
EEL, mm^2^	1.87 ± 0.33	1.64 ± 0.63	1.98 ± 0.48	1.39 ± 0.39	0.37
IEL, mm^2^	1.32 ± 0.24	1.15 ± 0.55	1.58 ± 0.24	0.99 ± 0.45	0.52
Lumen, mm^2^	1.18 ± 0.24	1.00 ± 0.55	1.32 ± 0.30	0.92 ± 0.43	0.58
Media, mm^2^	0.55 ± 0.10	0.50 ± 0.11	0.59 ± 0.18	0.40 ± 0.08	0.21
Neointimal area, mm^2^	0.15 ± 0.06	0.14 ± 0.04	0.11 ± 0.05	0.06 ± 0.02	0.085
Neointimal thickness, mm	0.10 ± 0.011	0.069 ± 0.022	0.066 ± 0.018	0.053 ± 0.003	0.005
Percent area stenosis, %	10.88 ± 4.52	9.99 ± 3.78	7.92 ± 3.84	6.80 ± 2.74	0.45
**Histological Analysis**					
Injury	1.00 ± 0.41	0.75 ± 1.19	1.13 ± 1.32	0.50 ± 0.41	0.74
EC Score	0.25 ± 0.50	0.00 ± 0.00	0.25 ± 0.50	1.50 ± 0.58	0.013
Inflammation	0.25 ± 0.50	0.00 ± 0.00	0.50 ± 0.58	0.25 ± 0.50	0.86
SMC Loss	0.00 ± 0.00	0.00 ± 0.00	0.25 ± 0.50	1.00 ± 0.82	0.081

Abbreviations: EEL—external elastic lamina, IEL—internal elastic lamina, EC—endothelial cell, SMC—smooth muscle cell.
